# Prevalence of piroplasms in small ruminants in North-West Tunisia and the first genetic characterisation of *Babesia ovis* in Africa

**DOI:** 10.1051/parasite/2014025

**Published:** 2014-05-22

**Authors:** Mohamed Ridha Rjeibi, Mohamed Gharbi, Moez Mhadhbi, Wiem Mabrouk, Boutheïna Ayari, Ines Nasfi, Mohamed Jedidi, Limam Sassi, Mourad Rekik, Mohamed Aziz Darghouth

**Affiliations:** 1 Laboratoire de Parasitologie, Université de la Manouba, École Nationale de Médecine Vétérinaire de Sidi Thabet 2020 Sidi Thabet Tunisia; 2 International Centre for Agricultural Research in the Dry Areas (ICARDA) PO Box 950764 Amman 11195 Jordan

**Keywords:** Small ruminants, Haemopathogen, *Babesia ovis*, *Theileria ovis*, PCR, Tunisia

## Abstract

In this study, the prevalence of piroplasms in sheep and goats was assessed with Giemsa-stained blood smear examination, PCR and nested PCR-restriction fragment length polymorphism (RFLP) to identify *Babesia* and *Theileria* species, respectively, in 338 small ruminants (172 sheep and 166 goats) from three sites in North-West Tunisia during the 2011 summer season. The overall infection prevalence of piroplasms in Giemsa-stained blood smears was 3.2% (11/338), with a parasitaemia ranging from 0.01 to 0.05%. PCR detected two species, namely *Babesia ovis* (in sheep and goats) and *Theileria ovis* (in sheep), with an overall prevalence of 16.3%. The molecular prevalence of *B. ovis* was significantly higher in sheep than in goats (17.4% and 9%, respectively, *p* = 0.034). The same trend was observed for *T. ovis* in sheep and goats (5.8% and 0%, respectively, *p* = 0.004). Comparison of the partial sequences of the 18S ssu rRNA gene revealed 100% similarity amongst *Babesia* from sheep and goats. The single *Theileria* sequence in this study showed 100% similarity to *T. ovis*. A high similarity with all the blasted genotypes was reported for *Theileria* and *Babesia* sequences. This is the first molecular detection of *B. ovis* and genetic characterisation of small ruminants’ piroplasms in Africa.

## Abstract (fr)

Dans cette étude, la prévalence des piroplasmes chez les ovins et les caprins a été évaluée par la technique de la coloration au Giemsa, la PCR et la PCR nichée et l’étude du polymorphisme des fragments de restriction (RFLP) pour identifier respectivement les espèces de *Babesia* et *Theileria* sur 338 petits ruminants (172 ovins et 166 caprins) provenant de trois sites au nord-ouest de la Tunisie pendant la saison estivale 2011. La prévalence globale de l’infection par les piroplasmes par la technique de la coloration au Giemsa était de 3,2 % (11/338) avec une parasitémie variant entre 0,01 et 0,05 %. La PCR a détecté deux espèces, *Babesia ovis* (ovins et caprins) et *Theileria ovis* (ovins), avec une prévalence globale de 16,3 %. La prévalence moléculaire de l’infection par *B. ovis* était significativement plus élevée chez les ovins que les caprins (respectivement 17,4 % et 9 %, *p* = 0,034). La même tendance a été observée pour *T. ovis* chez les ovins et les caprins (respectivement 5,8 % et 0 %, *p* = 0,004). La comparaison des séquences partielles du gène 18S ARNr a révélé une homologie de 100 % entre les séquences de *Babesia* des ovins et des caprins. La seule séquence de *Theileria* de cette étude a montré une homologie de 100 % avec *T. ovis*. Une grande homologie avec les génotypes étudiés par BLAST a été retrouvée pour les séquences de *Theileria* et de *Babesia*. Ceci est la première détection moléculaire de *B. ovis* et la première caractérisation génétique des piroplasmes de petits ruminants en Afrique.

## Introduction

There are over 1.07 billion sheep in the world; 27% of them are located in Africa [15]. Sheep are amongst the major economically important livestock in Tunisia, with a total population of 6.5 million sheep; they play an important role in the livelihood of resource-poor farmers. The goat population is lower; it was estimated at 1.5 million head in Tunisia [28].

In North Africa, small ruminants are exposed to several health problems, such as abortive diseases (brucellosis, border disease, toxoplasmosis, salmonellosis, campylobacteriosis) [19], and gastrointestinal and respiratory helminths [2–4]. Moreover, the stock owners face extreme climatic conditions with a very long dry period, leading to dramatic decreases in food resources’ quality and quantity. Several inputs are expensive such as diesel oil, imported concentrate and drugs, leading to a weakening of the financial assets of the small-scale farmers. In addition, climate change, in particular global warming, is further exacerbating the fragile environment where the animals are thriving. Many endemic pathogens are neglected by stock owners since they do not cause significant symptoms or financial losses. Some of them are highly prevalent in animal populations; they cause small but persistent losses, and when cumulated all together they generate huge losses to the farmers. Piroplasms are small ruminants’ neglected infections. Several species of *Babesia* (*B. ovis, Babesia motasi, Babesia crassa* and *Babesia* sp. Xinjiang) have been described in small ruminants; amongst them, *B. ovis* and *B. motasi* are believed to be causative agents of babesiosis [25, 30, 35, 41].

Recently, two studies were carried out in South Africa and northern Ethiopia; they showed the presence of several species of *Theileria* in domestic small ruminants. In northern Ethiopia, two *Theileria* species were recently reported in small ruminants, namely *T. ovis* and *Theileria separata*, whereas no *Babesia* spp. was found [18]. In South Africa, four piroplasm species were found in sheep, namely *T. ovis*, *T. separata*, *Theileria bicornis* and *Theileria* sp. (sable) [8].

Screening piroplasm-infected small ruminants can be carried out either by blood smear examination, which is a rapid, cheap and easy but not sensitive technique, or by PCR, which is sensitive but expensive. The combination in series of these two techniques can represent a powerful tool for screening animals infected with piroplasms.

We describe herein an epidemiological survey carried out on small ruminants’ piroplasm infection in a humid region of Tunisia where production systems are low-input and extensive. This epidemiological study was followed by a genetic comparison of the Tunisian small ruminant piroplasms with others from different regions in the world.

## Materials and methods

### Study area

**Figure 1.**
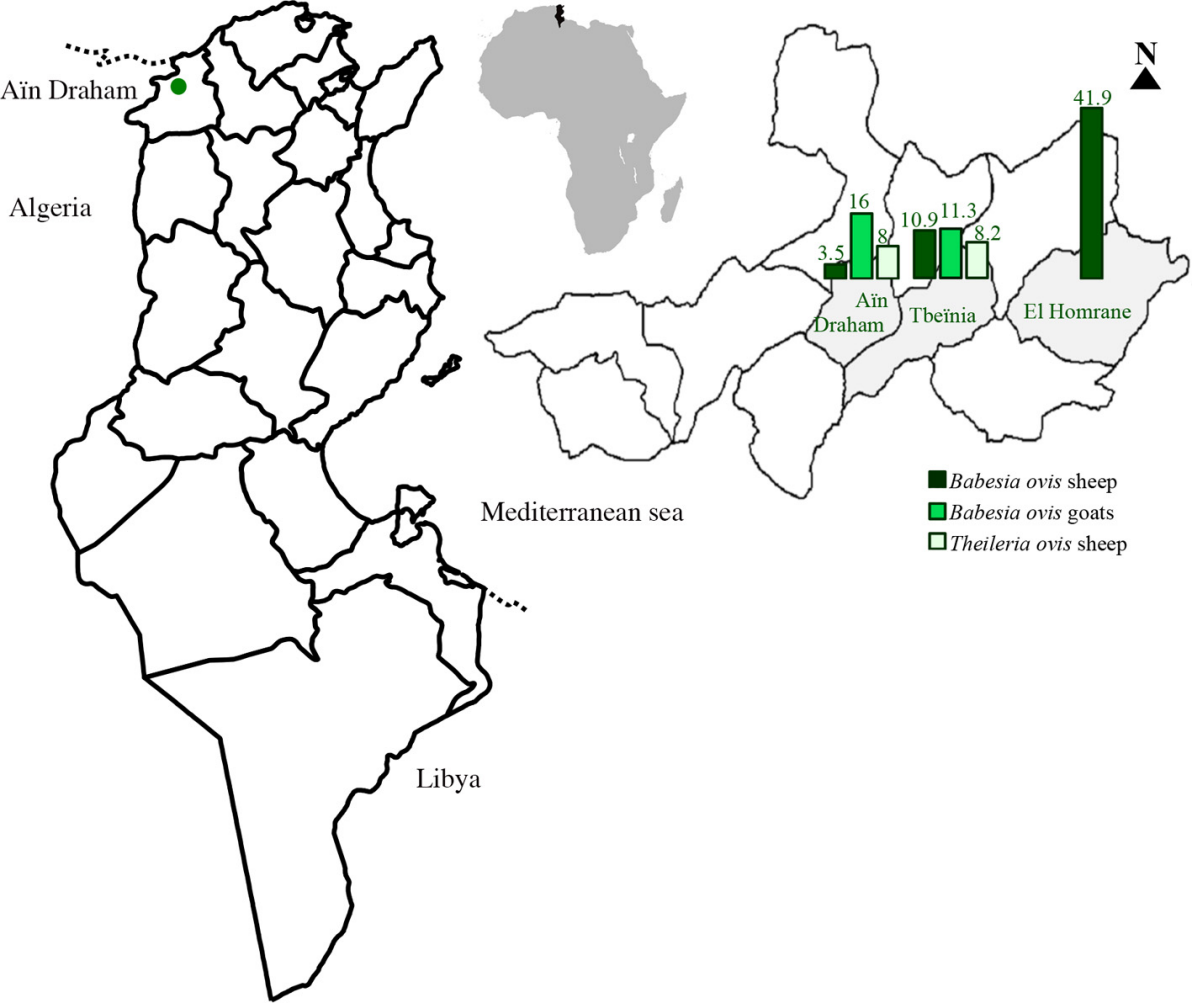
Sheep and goat PCR infection prevalence of *Babesia ovis* and *Theileria ovis* in the Aïn Draham locality (North-West Tunisia).

The present study was carried out in three sites of Aïn Draham (district of Jendouba, North-West Tunisia; Fig. 1). This region has an altitude ranging between 305 and 680 m; it is humid, with a mean annual rainfall of 1300 mm, and dry during the summer season. The mean minimal temperature is 8.1 °C in January, whilst the mean maximum temperature is 30 °C in August (National Institute of Meteorology, Tunisia).

### Animals and samples

During the 2011 summer season (June and July), clinically healthy sheep (*n* = 172) and goats (*n* = 166) were included in the survey from 31 randomly selected small ruminant flocks. The sheep belong to two breeds, namely Queue Fine de l’Ouest (60.4%) and Barbarine sheep (39.5%). The goats were of the local population (86.7%) and Damascus genotypes (13.2%). A full description of the animals’ breeds was reported by Rekik et al. [33]. The animals graze daily on spontaneous vegetation. During summer, it is occasionally supplemented by bran, barley and concentrate. Based on dentition, animals were ranked into three age groups: less than one year, between one and two years and more than two years. All the animals included in the current survey were examined for ticks, which were collected in labelled tubes containing 70% ethanol and identified under a stereomicroscope based on the key of Walker et al. [42]. Three tick infestation indicators were determined:

Infestation prevalence (%) = 100 × (number of infested animals/total number of animals).
Infestation intensity = number of ticks/number of infested animals.
Abundance = number of ticks/total number of animals.

Blood samples were collected in EDTA tubes from the jugular vein of each animal. Giemsa-stained blood smears were examined under a microscope with immersion oil at 1000× magnification for the presence of piroplasms. For each slide, 50 microscopic fields were examined.

### Polymerase chain reaction and PCR-RFLP

DNA was extracted from 300 μL of blood using the Wizard^®^ Genomic DNA purification kit (Promega, Madison, USA) according to the manufacturer’s instructions; it was stored at −20 °C until used. Catch-all primers (RLB-F and RLB-R) which detect both *Theileria* spp. and *Babesia* spp. piroplasms were used [20] (Table 1). Forty PCR cycles were performed with a thermocycler (ESCO Swift MaxPro) in a total reaction volume of 25 μL. Five microlitres of each positive sample were amplified by 35 PCR cycles using primers detecting *B. ovis* (Bbo-F and Bbo-R) [5] (Table 1). A nested PCR detecting specific *Theileria* DNA of the 18S ssu rRNA gene was performed. The primary PCR consisted of 25 cycles realised with 20 pg of outer primers (Thei F1 and Thei R1) in 30 μL volume. One microlitre of each PCR product was used as a template in a 30-cycle nested PCR in the same mixture as the primary PCR with two different primers (Thei F2 and Thei R2) [21] (Table 1).

**Table 1.** primers used for detection by PCR of *Babesia ovis* and *Theileria* spp. from sheep and goats in this study.

Primer specificity	Target Gene	Name	Type	Primers 5′–3′	Product size (bp)	Reference
*Catch-all*	18S rRNA	RLB-F	Forward primer	GAGGTAGTGACAAGAAATAACAATA	460–520	Gubbels et al. [20]
		RLB-R	Reverse primer	TCTTCGATCCCCTAACTTTC		
*B. ovis*	18S rRNA	Bbo-F	Forward primer	TGGGCAGGACCTTGGTTCTTCT	549	Aktas et al. [5]
		Bbo-R	Reverse primer	CCGCGTAGCGCCGGCTAAATA		
*Theileria* spp.	18S rRNA	Thei F1	Forward primer	AACCTGGTTGATCCTGCCAG	1700	Heidarpour Bami et al. [21]
		Thei R1	Reverse primer	AAACCTTGTTACGACTTCTC		
	18S rRNA	Thei F2	Forward primer	TGATGTTCGTTTYTACATGG	1417–1426	Heidarpour Bami et al. [21]
		Thei R2	Reverse primer	CTAGGCATTCCTCGTTCACG

**Table 2.** *Theileria ovis* and *Babesia ovis* infection prevalence and intensity in sheep and goats by Giemsa-stained blood smears and PCR in Aïn Draham, North-West Tunisia.

Species	Epidemiological indicator	Technique	Sheep	Goats	95% CI^c^	*p* value
*B. ovis*	Infection prevalence = 100 × (number of positive blood samples/number of examined blood samples) (%)	MO^a^	5/172 (2.9)	4/166 (2.4)	[0.28; 5.48]	0.77
		PCR	30/172 (17.4)	15/166 (9.04)	[1.05; 4.34]	0.022*
	Infection intensity = 100 × (number of positive red blood cells/number of examined red blood cells)	MO	0.015	0.031	[0; 0.004]	0.102
*T. ovis*	Infection prevalence = 100 × (number of positive blood samples/number of examined blood samples) (%)	MO	2/172 (1.16)	0/166 (0)	NA^b^	0.16
		PCR	10/172 (5.81)	0/166 (0)	NA	0.001*
	Infection intensity = 100 × (number of positive red blood cells/number of examined red blood cells) (%)	MO	0.022	0	[0; 0.005]	0.105

a MO – Microscopy observation.

b NA – not applicable.

c CI – 95% Confidence interval.

* Significant test.

In order to differentiate *Theileria annulata, Theileria lestoquardi* and *T. ovis*, restriction fragment length polymorphism (RFLP) was performed with *HpaII* and *HaeII* restriction enzymes (Fermentase) [21]. Ten microlitres of the PCR product were mixed with 2 μL of 10*×* enzyme buffer and 10 U of the restriction enzyme, then incubated at 37 °C for 2 h (Table 3).

**Table 3.** RFLP pattern of different *Theileria* species after *HpaII* and *HaeII* digestion (Heidarpour Bami et al. [21]).

*Theileria* species	Digestion products obtained by *HpaII* (bp)	Digestion products obtained by *HaeII* (bp)
*Theileria ovis*	856, 326, 204 and 39	1131 and 295
*Theileria annulata*	1178, 106, 94 and 39	No digestion
*Theileria lestoquardi*	900, 278, 106, 94 and 39	No digestion

### DNA sequencing and phylogenetic analysis

Three selected PCR products were purified with the Wizard SV gel and PCR clean-up system (Promega, Madison, USA) according to the manufacturer’s instructions. The products were sequenced in both directions with the same primers as for PCR. A conventional Big Dye Terminator cycle sequencing ready reaction kit (Applied Biosystems, Foster City, CA, USA) with an ABI3730XL automated DNA sequence was used. The chromatograms were evaluated with ChromasPro software (version 1.7.4). The MEGA 5.1 program was used to perform multiple sequence alignments [39]. The sequences were compared with the GenBank database by a nucleotide sequence homology search carried out at the network server of the National Centre for Biotechnology Information (NCBI) using BLAST. The sequence of the 18S ssu rRNA genes of *B. ovis* from sheep (BOTNSHAD01) and goats (BOTNGTAD01) and *T. ovis* from sheep (TOTNSHAD01) identified in the present survey were deposited in GenBank under Accession Nos. KJ192344, KF723611, KJ192344, KF723612 and KJ192344, KF723613, respectively. Phylogenetic trees were constructed by the neighbour-joining method [34]. The percentage of replicate trees in which the associated taxa clustered together in the bootstrap test (1100 replicates) was shown next to the branches [16]. The evolutionary distances were computed using the Tamura-Nei method [38] and are in the units of the number of base differences per site. Evolutionary analyses were conducted with MEGA 5.1 software (Fig. 3).

### Statistical analyses

The infection prevalence percentages were compared using Epi Info 6 [13]. In order to consider any confusion factor, a chi-square Mantel-Haenszel test was performed. A probability less than 0.05 was used as a threshold for statistical significance [37]. The concordance between PCR and blood smears was estimated with a kappa test [40].

## Results

Only 24 and 15 ticks all belonging to *Rhipicephalus turanicus* were collected from sheep and goats, respectively. The overall prevalence of tick infestation was 8.87% (30/338), the intensity (1.3) and the abundance (0.11). There were no statistically significant differences in piroplasm prevalence in tick-infested and noninfested animals (*p* > 0.05). Our study showed that tick infestation prevalence was significantly higher in Damascus goats than local ones (*p* < 0.05). This difference was not reported in sheep (*p* > 0.05). The overall infection prevalence of piroplasms was 3.25% (11/338) and the overall mean parasitaemia was 0.011% (range: 0.01%–0.05%).

**Figure 2.**
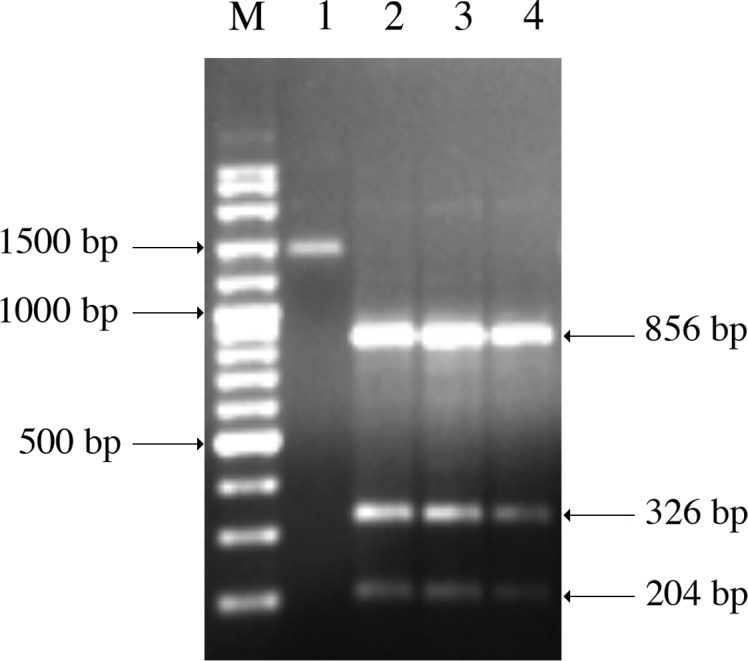
Digestion pattern of nested *Theileria* spp. 18S ssu rRNA gene PCR products by *HpaII*. Lane M: 100 bp ladder; lane 1: *Theileria* spp. DNA; lanes 2–4: *Theileria ovis* (856, 326 and 204 bp).

All positive blood smears were positive by nested PCR. The enzymatic digestion profile by *HpaII* showed that all the PCR products belong to *T. ovis* species (10/338) (Fig. 2). A total number of nine and two animals were exclusively infected by *Babesia* spp. and *Theileria* spp. in blood smears, respectively; no animals were coinfected by both parasites (Table 2). Fifty-five samples were positive for *T. ovis* and *B. ovis* by PCR. There is a moderate concordance between PCR and blood smears for *T. ovis* and *B. ovis* in sheep and *B. ovis* in goats (*κ* = 0.32, 0.25 and 0.4, respectively).

The molecular prevalence of *B. ovis* and *T. ovis* was higher in sheep than in goats (*p* = 0.034 and *p* = 0.004, respectively). There was no difference in the molecular prevalence of *B. ovis* and *T. ovis* for different age categories. The *B. ovis* infection rate in Barbarine sheep was higher than in Queue Fine de l’Ouest sheep (*p* < 0.05), whilst it was significantly higher in Damascus than local goats (*p* < 0.05), and no difference was detected in *T. ovis* prevalence in both hosts (*p* > 0.05) (Table 4). Infection prevalence of *B. ovis* varies in localities for sheep and goats (*p* < 0.05), whilst no difference was recorded in *T. ovis* (*p* > 0.05) (Table 4). The infection rate by *T. ovis* was significantly higher in females than in males (*p* < 0.05).

**Table 4.** Association between the presence of sheep and goat piroplasms and different parameters based on PCR.

Species	Parameter		*Babesia ovis*		*Theileria ovis*	
			Positive/examined (%)	OR [95% CI]	Positive/examined (%)	OR [95% CI]
Sheep	Gender	Female	23/126 (18.2)	0.9 [0.27; 2.96]	10/126 (7.9)*	NA^a^
		Male	7/46 (15.2)		0/46 (0)	
	Breed	Barbarine	18/68 (26.5)	2.76 [1.15; 6.61]*	5/68 (7.3)	1.57 [0.38; 6.58]
		QFO^b^	12/104 (11.5)		5/104 (4.8)	
	Age group	<1 year	4/21 (19)	3.37 [0.55; 21.81]	3/21 (14.3)	3.67 [0.44; 34.93]
		1 to 2 years	3/46 (6.5)		2/46 (4.3)	
		>2 years	23/105 (21.9)	4.02 [1.06; 17.88]*	5/105 (4.8)	1.1 [0.18; 8.55]
	Locality	Aïn Draham	1/28 (3.6)		2/28 (7.1)	NA
		Tbeïnia	11/101 (10.9)	3.3 [0.41; 71.41]	8/101 (7.9)	
		El Homrane	18/43 (41.9)	19.44 [2.4; 419.3]**	0/43 (0)	
Goats	Gender	Male	4/40 (10)	1.39 [0.29; 6.96]	0/40 (0)	NA
		Female	11/126 (8.7)		0/126 (0)	
	Breed	Damascus	6/22 (27.3)	5.63 [1.53; 20.48]*	0/22 (0)	NA
		Local	9/144 (6.2)		0/144 (0)	
	Age group	<1 year	0/18 (0)	NA	0/18 (0)	NA
		1 to 2 years	6/42 (14.3)		0/42 (0)	
		>2 years	9/106 (8.5)		0/106 (0)	
	Locality	Aïn Draham	4/25 (16%)*	NA	0/25 (0)	NA
		Tbeïnia	11/97 (11.3%)		0/97 (0)	
		El Homrane	0/44 (0%)		0/44 (0)	
Overall	Gender	Female	34/252 (13.5%)	0.67 [0.26; 1.6]	10/252 (4)	NA
		Male	11/86 (12.8%)		0/86 (0)	
	Age group	<1 year	4/39 (10.2%)		3/39 (7.7)	3.58 [0.46; 32.27]
		1 to 2 years	9/88 (10.2%)	1 [0.26; 4.16]	2/88 (2.3)	
		>2 years	32/211 (15.2%)	1.56 [0.59; 5.58]	5/211 (2.4)	1.04 [0.18; 7.93]
	Locality	Aïn Draham	5/53 (9.4%)		2/53 (3.8)	NA
		Tbeïnia	22/198 (11.1%)	1.2 [0.4; 3.83]	8/198 (4)	
		El Homrane	18/87 (20.7%)	2.5 [0.8; 8.32]	0/87 (0)	

* 0.001 ≤ *p* < 0.05.

** *p* < 0.001.

a NA – not applicable.

b Queue Fine de l’Ouest.

**Figure 3.**
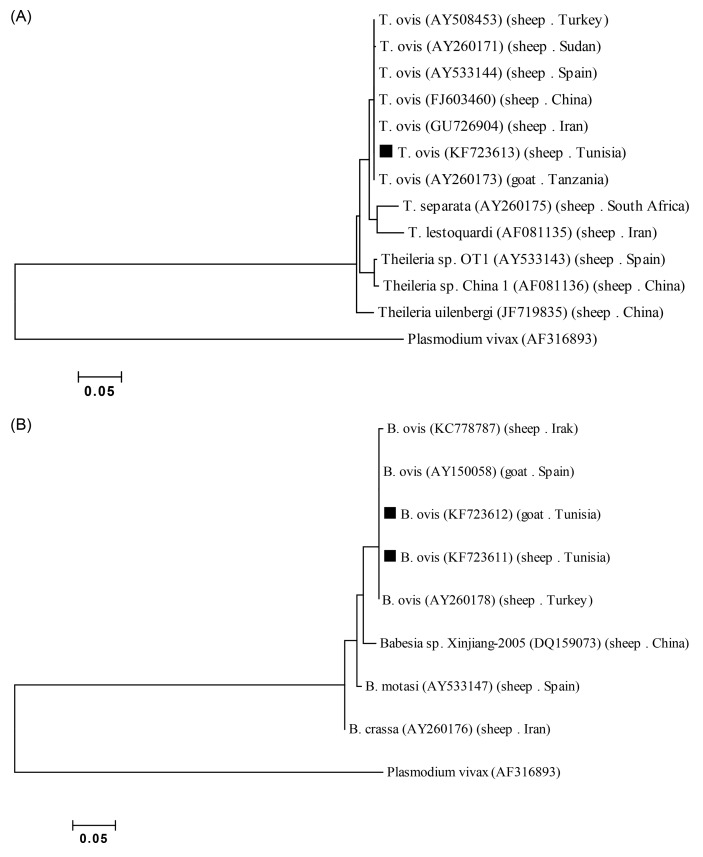
The tree was constructed using the neighbour-joining method [34]. The percentage of replicate trees in which the associated taxa clustered together in the bootstrap test (1100 replicates) is shown next to the branches [16]. The evolutionary distances were computed using the Tamura-Nei method [38] and are in the units of the number of base differences per site. Evolutionary analyses were conducted in MEGA5.1 [39]. GenBank accession numbers are given in parentheses. Species described in this study are indicated with a black square. (A) Partial sequence 18S ssu rRNA gene phylogenetic tree of the species identified in this survey and the main small ruminants’ *Theileria* species. (B) Partial sequence 18S ssu rRNA gene phylogenetic tree of the species identified in this survey and the main small ruminants’ *Babesia* species.

One *B. ovis* amplicon from sheep, another from goats and one *Theileria* from sheep were randomly chosen for genetic analysis. The comparison of the 18S ssu rRNA *B. ovis* sequence (509 bp length) revealed 100% homology between the two *Babesia* genotypes. The two amplicons from sheep and goats (509 bp) shared 100, 99.8, 99.6 and 99.2% homology with the recently reported sequences for the 18S ssu rRNA gene of *B. ovis* from Spain (KJ192344, AY150058), Turkey (KJ192344, AY260178), Iraq (KJ192344, KC778787) and eastern Turkey (KJ192344, AY998124), respectively (Fig. 3B). The *Theileria* amplicon (742 bp length) showed 100, 99.8, 99.8, 99.8, 99.8 and 99.7% homology with *T. ovis* from Iran (KJ192344, GU726904), China (KJ192344, FJ603460), Spain (KJ192344, AY533144), Tanzania (KJ192344, AY260173), Turkey (KJ192344, AY508458) and Sudan (KJ192344, AY260171), respectively (Fig. 3A).

A phylogenetic tree of *Theileria* and *Babesia* was constructed from the 18S ssu rRNA gene sequences of our amplicons and those available in GenBank. The first phylogenetic tree consists of all *Theileria* species; the *T. ovis* sequence (742 bp) described herein forms a well-supported clade with all the studied *T. ovis*, whereas the other *Theileria* species belonged to different clades such as *T. lestoquardi* and *T. separata* (Fig. 3A). Concerning *Babesia* sequences (509 bp), the phylogenetic analysis showed evidence of four monophyletic clades, one consisting of *B. ovis* and the others *B. crassa*, *B. motasi* and *Babesia* sp. Xinjiang-2005, with well-supported separation amongst them. The two *B. ovis* sequences described in this study formed a well-supported clade with the other *B. ovis* sequences clearly distinct from *B. motasi*, *B. crassa* and *Babesia* sp. Xinjiang-2005 (Fig. 3B).

## Discussion

Babesiosis is a tick-borne disease causing low but persistent losses with high prevalence of carrier state infection in small ruminants, thereby resulting in high economic losses in several tropical and subtropical regions [1, 27, 41]. Our survey samplings were performed during the summer season, since both *T. ovis* and *B. ovis* are transmitted by *Rhipicephalus bursa and R. turanicus*, which have a vernal activity [9, 12].

The diagnosis of piroplasm infections is mainly performed by microscopic examination of Giemsa-stained blood smears. However, this method has a low sensitivity and requires expertise because these parasites have similar morphology and therefore, different species may be confused. The detection of *Babesia* infection in carrier animals by DNA amplification is a powerful tool for epidemiological investigations, since it is more sensitive and specific than Giemsa-stained blood smears [7, 11].

In the present study, the molecular prevalence of *B. ovis* in small ruminants was significantly higher than Giemsa-stained blood smear examination (2.66%); the latter technique does not detect carrier animals with very low parasitaemia. This result is consistent with previous reports about *B. ovis* [6] and *Babesia* spp. [30] in Turkey and Iran, respectively. The overall parasitaemia ranged from 0.01% to 0.05%. In similar studies, other findings showed that *B. ovis*-infected sheep had low parasitaemia (0.01%–0.1%) [29, 32].

*Babesia ovis* molecular prevalence was significantly higher in sheep (30/172, 17.44%) than goats (15/166, 9.04%) (*p* = 0.022). It was stated that *B. ovis* induces symptoms more frequently in sheep than goats [17]. In Turkey, sheep *B. ovis* infection prevalence was higher than in goats (10.66 and 1%, respectively) [6]; the same trend was observed in Pakistan (50% and 24%, respectively) [23], contrary to another survey carried out in Turkey, where no significant difference was reported between sheep and goats’ infection prevalence (2.9% and 2%, respectively) [22].

The highest *B. ovis* infection rate was observed in Barbarine sheep compared with the Queue Fine de l’Ouest breed (26.5% and 11.5%, respectively) (*p* < 0.05) but in goats, the infection prevalence was higher in crossbred animals compared with animals of the local population (27.3% and 6.2%, respectively) (*p* < 0.05). For sheep, this can be explained by the fact that animals of the Barbarine breed are not in their natural environment, which is the steppe of dry land in central Tunisia. In goats, the results may refer to a higher genetic resistance of local breeds to piroplasms in comparison with exotic breeds.

No significant association was observed between the animals’ ages and *B. ovis* infection prevalence, confirming the presence of an endemic stability state, as reported by others [6, 31, 32]. This is contrary to the findings in Pakistan [23], where the prevalence in animals aged less than one year was higher. No difference was detected in *B. ovis* prevalence between males and females (*p* > 0.05); our results are contradictory to other surveys reporting that the prevalence in male sheep and goats was higher than females [23]. The association between tick burdens and piroplasm prevalence was statistically not significant (*p* > 0.05); our results do not coincide with other findings that reported the presence of a positive correlation between tick burden and infection prevalence [5, 14, 24]. The two *B. ovis* sequences (from sheep and goat) had 100% similarity for the 18S ssu rRNA gene. They were also identical to the Spanish sequence (KJ192344, AY150058) [10] and had a high genetic homology with all *B. ovis* deposited sequences in GenBank, namely eastern Turkey (99.2%) (KJ192344, AY998123), Iraq (99.6%) (KJ192344, KC778787) and Central Turkey (99.8%) (KJ192344, AY260178) [36]. Contrary to reports in Tunisia [26], Pakistan [24] and Ethiopia [18], which detected *T. ovis* in sheep and goats, this species was only present in sheep. Only females were infected by *T. ovis*; this result disagrees with others who reported the presence of the parasite in both sexes [24]. Unlike other surveys, we did not find any significant variation between different ages and breed groups [26].

*Theileria ovis* from sheep showed lower genetic diversity. The Iranian sequence (KJ192344, GU726904) [43] had a 100% homology with our sequences; all the *T. ovis* sequences from three continents (Africa, Europe and Asia) were clustered together in a big clade clearly distinct from *Theileria* sp. China (KJ192344, AF081136) and *T. lestoquardi* (KJ192344, AF081136), which are pathogenic for small ruminants. As far as could be ascertained from accessible published works, there are no published reports on small ruminants’ *B. ovis* infection in Africa. Further studies are needed to improve our knowledge on small ruminants’ ticks and tick-borne pathogen epidemiology in North Africa and to explore the role of these piroplasms in sheep and goat pathology.
